# The Druggist’s General Receipt Book

**Published:** 1853-05

**Authors:** 


Art. XIV.—*The Druggist's General Receipt Book;* Comprising a copious Veterinary Formulary, and Table of Veterinary Materia Medica; numerous recipes of patent and proprietary medicines, druggist's nostrums, etc: Perfumery and Cosmetics; beverages, dietetic articles, and condiments; trade chemicals, &c. With an appendix of useful tables. By Henry Beasley. Second American from the last London edition, corrected and enlarged. Philadelphia: Lindsay & Blakiston: 1853. 12mo. pp. 472.

The character of this compendious little volume will be seen by a glance at the title-page; it is a receipt book, in which matter relating to almost every topic pertaining to pharmacy may be found, and much, doubtless, which every man may occasionally find it convenient to understand. We hope none of our readers will be deterred from purchasing it by the mention of "patent medicines and druggist's nostrums" in the title-page. It is well enough to know what is the composition of these articles, and this, so far as his acquaintance enables him, Mr. Beasley has given in his book. We believe that druggists will find it a very valuable hand-book, and that other than druggists may derive much useful information from it.

